# ImmunoPET imaging of TIGIT in the glioma microenvironment

**DOI:** 10.1038/s41598-024-55296-y

**Published:** 2024-03-04

**Authors:** Sarah R. Vincze, Ambika P. Jaswal, Stephen C. Frederico, Michal Nisnboym, Bo Li, Zujian Xiong, ReidAnn E. Sever, Chaim T. Sneiderman, Mikayla Rodgers, Kathryn E. Day, Joseph D. Latoche, Lesley M. Foley, T. Kevin Hitchens, Robin Frederick, Ravi B. Patel, Costas G. Hadjipanayis, Itay Raphael, Jessie R. Nedrow, W. Barry Edwards, Gary Kohanbash

**Affiliations:** 1 Department of Neurological Surgery, University of Pittsburgh School of Medicine, Pittsburgh, PA USA; 2 Department of Neurology, Tel-Aviv Sourasky Medical Center, Tel-Aviv University, Tel-Aviv, Israel; 3 In Vivo Imaging Facility, University of Pittsburgh Medical Center, UPMC Hillman Cancer Center, Pittsburgh, PA USA; 4 Department of Radiology, University of Pittsburgh Medical Center, Pittsburgh, PA USA; 5 Department of Biochemistry, University of Missouri, Columbia, MO USA; 6 Department of Immunology, University of Pittsburgh, Pittsburgh, PA USA; 7 Department of Neurobiology, University of Pittsburgh, Pittsburgh, PA USA

**Keywords:** TIGIT, Brain tumor, Immunotherapy, Glioma, Immunosuppression, Cancer imaging, CNS cancer, Imaging the immune system, Immunotherapy, Tumour immunology

## Abstract

Glioblastoma (GBM) is the most common primary malignant brain tumor. Currently, there are few effective treatment options for GBM beyond surgery and chemo-radiation, and even with these interventions, median patient survival remains poor. While immune checkpoint inhibitors (ICIs) have demonstrated therapeutic efficacy against non-central nervous system cancers, ICI trials for GBM have typically had poor outcomes. TIGIT is an immune checkpoint receptor that is expressed on activated T-cells and has a role in the suppression of T-cell and Natural Killer (NK) cell function. As TIGIT expression is reported as both prognostic and a biomarker for anti-TIGIT therapy, we constructed a molecular imaging agent, [^89^Zr]Zr-DFO-anti-TIGIT (^89^Zr-αTIGIT), to visualize TIGIT in preclinical GBM by immunoPET imaging. PET imaging and biodistribution analysis of ^89^Zr-αTIGIT demonstrated uptake in the tumor microenvironment of GBM-bearing mice. Blocking antibody and irrelevant antibody tracer studies demonstrated specificity of ^89^Zr-αTIGIT with significance at a late time point post-tracer injection. However, the magnitude of ^89^Zr-αTIGIT uptake in tumor, relative to the IgG tracer was minimal. These findings highlight the features and limitations of using ^89^Zr-αTIGIT to visualize TIGIT in the GBM microenvironment.

## Introduction

Malignant gliomas are the most common primary central nervous system malignancy in adults with glioblastoma (GBM) being the most common and most lethal^1^. Standard of care treatment for GBM consists of surgical resection, chemotherapy, and radiation therapy. However, the median overall survival for patients diagnosed with GBM remains < 2 years^2^. There have been many preclinical and clinical studies evaluating immunotherapy for patients with GBM, including with immune checkpoint inhibitors (ICI), adoptive cell therapies, and cancer vaccines; however, while some are promising, these efforts have generally shown limited efficacy as of this time.

A major challenge surrounding the use of immuno-therapeutics in the treatment of brain tumors is differentiating which patients are strong candidates versus those that are not^3^. Additionally, evaluating the efficacy of immunotherapy for brain tumors is challenging as few measures exist for determining therapeutic efficacy beyond increased patient survival, and observing disease regression via conventional imaging studies. Previous studies in non-CNS cancers have demonstrated that PD-L1 (programmed death ligand 1) expression is associated with a patient response to PD1/PD-L1 ICI therapy^4,5^. Hettich et al. determined that PD-L1 expression by positron emission tomography (PET) imaging was associated with response to anti-PD-L1 therapy in mice bearing melanoma^6^. Given the significant heterogeneity in the tumor microenvironment (TME) of GBM, imaging methodologies to assess immune checkpoints in GBM, for patient stratification, have significant potential.

T-cell immunoreceptor with Ig and ITIM domain (TIGIT) is an immune checkpoint receptor that is expressed on activated T-cells, Natural Killer (NK) cells, and Regulatory T-cells (Tregs). TIGIT binds with high affinity to CD155 and with low affinity to CD112 (PVRL2; nectin-2)^7^. CD155 and CD112 are expressed in the TME by antigen presenting cells (APCs) and by tumor cells. TIGIT binding to CD155 can inhibit activated T-cells and NK cell function^7^. CD155 can also bind with CD226, expressed on many immune cells including T and NK cells, as an immune stimulatory signal, however, TIGIT has a stronger affinity for CD155, compared with CD226^8,9^. Prior studies have shown that TIGIT is expressed on T- and NK cells present in murine and human GBM^9,10^. Given that the high expression of TIGIT in GBM is associated with worse patient prognosis, TIGIT is reported as a promising, potential immune-therapeutic target for GBM patients^9,10^.

Here, we describe the feasibility of employing an anti-TIGIT antibody radiolabeled with the PET radioisotope Zirconium-89 (^89^Zr-αTIGIT) for GBM PET imaging. Shaffer et al. previously demonstrated that anti-TIGIT antibody allowed PET imaging of tumor infiltrating cells in a preclinical immunocompetent mouse melanoma model^11^. Another study reported by Wang et al. showed that ^68^Ga-GP12 is a promising peptide-based tracer for PET imaging of TIGIT expression^12^. Our study evaluates the potential and practicability of ^89^Zr-αTIGIT immunoPET to detect the presence of TIGIT in the TME of mice with orthotopic syngeneic GBM.

## Methods

### Murine glioma model

Murine C57BL/6-syngeneic GL261 glioma cells (obtained from the Division of Cancer Treatment and Diagnosis (DCTD) tumor repository of the National Cancer Institute (NCI) were cultured and prepared for stereotactic injection as previously reported^13^. Briefly, C57BL/6 mice (female, 5–8 weeks old; Jackson Laboratory) were anesthetized with isoflurane and placed on a stereotactic frame for tumor cell injection. Each mouse was injected with 1 × 10^5^ GL261 cells into the right caudate nucleus at + 2.5 mm medial/lateral and − 3.0 mm dorsal/ventral from the skull position of bregma using a micropump injector (World Precision Instruments). Mice with one or two week established tumors were used as indicated. All experiments were performed under an institutional approved protocol by the University of Pittsburgh Institutional Animal Care and Use Committee (IACUC; Approval # 22,101,912), and all experiments were carried out in accordance with the guidelines issued by the University of Pittsburgh IACUC. This study was carried out in compliance with the ARRIVE guidelines. Animals were inspected daily for health issues and were euthanized by CO_2_ asphyxiation followed by cervical dislocation. Criteria for euthanasia were based on an independent assessment by a veterinarian according to AVMA euthanasia guidelines.

### Flow cytometry

Spleen and tumor tissues were collected from GL261-bearing mice, and enzymatically processed into single-cell suspensions as previously described ^9,13^. For staining of GL261 cells, non-specific Fc binding was blocked using TruStain FcX™ (human or anti-mouse CD16/32, clone 93), for ex vivo spleen and tumor cells, staining was performed using anti-CD3 (clone 17A2), anti-CD11B (M1/70) and anti-TIGIT (clone 1G9); for in vitro GL261 staining, IgG (anti-mouse IgG1, clone MOPC-21), anti-TIGIT (anti-mouse TIGIT, clone 1G9), and anti-PD-L1 (anti-mouse CD274, clone 10F.9G2) were used. All staining antibodies were acquired from BioLegend. Flow cytometry data were collected using a LSR Fortessa (BD Biosciences) FlowJo software (Version 10.8.1). Two-way ANOVA with multiple comparison tests was used to assess statistical significance for all flow cytometry results.

### Histology

Tumors were harvested and fixed in 10% formalin (Fisher Scientific) for 24 h, then transferred to 70% ethanol. Further studies were performed post radioactivity decay. A 70–100% EtOH gradient was used to dehydrate the tissues over 12 h prior to clearance with histology-grade xylene (Leica) and paraffin embedding (Paraplast Plus, Leica). Next, the tissues were sectioned into 4 µm slices with a Leica RM2235 microtome, transferred onto Superfrost Plus slides (Fisher Scientific), and baked for 30–60 min at 60 °C. Antigen retrieval was performed using citrate and EDTA retrieval solutions at pH 6 and 9, respectively, and a decloaking chamber set to 120 °C. The slides were stained using an Autostainer Plus (Dako) with TBST rinse buffer (Dako). A CD3 antibody (polyclonal, Biocare Medical) was applied using a 1:400 dilution, while the TIGIT antibody (clone EPR26037-152, Abcam) was applied using a 1:100 dilution. For CD3 and TIGIT detection, a Mach 2 Rabbit polymer (Biocare Medical) and a Boost Rabbit HRP polymer (Cell Signaling, Danvers, MA) were used. The substrate used was 3,3, Diaminobenzidine + (Dako). The slides were then counterstained with Hematoxylin (Dako).

### Single-cell RNA sequencing (scRNAseq) analysis

ScRNAseq data was downloaded from a publicly available dataset, GSE182109^14^, on the GEO database. A total of 44 tumor specimens from 18 glioma patients [2 low-grade glioma (LGG), 11 newly diagnosed GBM (ndGBM), and 5 recurrent GBM (rGBM)] were analyzed. Seurat V4 was used to perform QC (Quality Checks), dimensionality reduction, and cell clustering. Specifically, the Seurat Feature Plot function and ggplot2 package were used to visualize TIGIT expression among glioma, immune, and stromal cell subsets. Reclustering of T-cells was performed using Seurat^15^ and T-cell subset identification was performed by ProjecTIL^16^. Color-coded cluster uniform manifold approximation and projections (UMAPs) were generated to illustrate TIGIT expression in all glioma cell types as well as expression stratified by glioma subtype—LGG or GBM (ndGBM and rGBM). Pairwise comparisons were assessed statistically using the Wilcoxon Rank-sum test performed by R.4.1.

### ^89^Zr-DFO-anti-TIGIT (^89^Zr-αTIGIT) production

InVivoMAb anti-mouse TIGIT (clone 1G9) and murine isotype control IgG1 (clone MOPC-21) were purchased from BioXcell. Deferoxamine (DFO) chelator (p-SCN-Bn-DFO) was purchased from Macrocyclics. Zirconium-89 (^89^Zr) was purchased from Washington University. The DFO-TIGIT conjugate was generated similar to a previously published protocol^17^. Briefly, a five-fold molar excess of p-SCN-Bn-DFO (13.3µL of 5 mM DFO-DMSO solution) was gradually added to a 2 mg/mL concentrated solution of anti-TIGIT antibody (1 mL), and sodium carbonate (0.1 M) was used to adjust the pH accordingly. Post 1 h incubation reaction mixture was purified using Cytiva 30 K centrifugal filters followed by PBS washes (3x). Following conjugation, radiolabeling was performed as previously described, with modifications, and was used to generate ^89^Zr-αTIGIT^18^. Specifically, 500 µCi ^89^Zr in 1 M oxalic acid (25 µL) and 1 M HEPES (175µL) were added per DFO-TIGIT solution (with a pH of 7–8), and the reactions were each incubated for 1 h at 37 °C. Parallel conjugation and radiolabeling reactions were carried out for IgG1 isotype control antibody. The radiochemical yield and purity of the tracer were determined by size exclusion column high-performance liquid chromatography (SEC-HPLC), performed on an Agilent 1260 infinity HPLC (Agilent Technologies) equipped with a Bio SEC-3 4.6 mm × 300 mm column. The UV–Vis 280 and 254 nm wavelengths were used to visualize TIGIT Ab and DFO elution, while a gamma radiation detector was used to visualize free ^89^Zr and ^89^Zr-αTIGIT. Free ^89^Zr in the radiolabeled product was removed by using a Cytiva 30 K centrifugal filter to achieve ≥ 95% radiopurity.

### PET imaging and biodistribution studies

Mice were injected intravenously (i.v.) with 100 µL of ^89^Zr-αTIGIT at a 2.3–2.5 MBq/25 µg dose; additionally, a group of mice were pre-treated 24 h prior with 250 µg of unconjugated anti-TIGIT. Static PET/CT imaging was performed on an Inveon small animal microPET/CT (Siemens Molecular Imaging) at 72-, 120- and 240-h following tracer injections. PET/CT images were co-registered and analyzed using Vivoquant (version 9); the SUV means were generated based on volume of interests (VOIs) for the tumor, normal brain (left), blood (vena cava), bone marrow, and heart, to evaluate changes in the tracer between mice over time. Biodistribution studies were performed following the last PET imaging time point, and select tissues were harvested, weighed, and measured. Tissue-associated radioactivity was detected by Wizard 2-Detector Gamma Counter (Perkin Elmer, model 2480) and analyzed as percent injected dose (%ID)/gram. The PET imaging and ex vivo biodistribution data was analyzed on GraphPad Prism (v10.1.2).

### TIGIT specificity studies

PET imaging and analysis were performed at 72-, 120- and 240-h post-injection of tracer with and without 10 × unconjugated anti-TIGIT (250 µg) to competitively inhibit tracer accumulation. An IgG1 isotype (InVivoMAb IgG1 mouse isotype, BioXcell; Clone MOPC-21) radiolabeled with zirconium-89, ^89^Zr-IgG1, was utilized as an irrelevant control. Mice with tumors 1-week post inoculation of GL261 cells (1-week tumors) were injected i.v. with either ^89^Zr-αTIGIT (2.9–3.1 MBq/100 μg) alone or ^89^Zr-IgG1 (2.9–3.3 MBq/100 μg). Additionally, a group of mice were pre-injected with unconjugated anti-TIGIT (625 μg) 24-h prior to ^89^Zr-αTIGIT to serve as a blocking control. SUV analysis was performed for tumor, normal brain, blood (vena cava), heart, and marrow.

### Stability studies

Stability studies of ^89^Zr-αTIGIT were assessed by incubating 2 µL aliquots of ^89^Zr-αTIGIT in mouse serum (a) or PBS (b), and at 72-, 120-, and 192-h. Post-incubation samples were analyzed by radio-iTLC. Percent stability was calculated utilizing the area under the curve divided by the total counts. Finally, stability was also confirmed utilizing radio-SEC-HPLC at 192 h in PBS.

### Binding assay

Streptavidin coated magnetic beads were used to assess the immunoreactivity of ^89^Zr-αTIGIT, based on prior published work ^19^. A 100-fold molar excess of biotinylated TIGIT antigen was incubated for one hour at room temperature with thorough agitation with the streptavidin beads. The beads were then washed (3x) before adding 0.01–0.03 MBq of ^89^Zr-αTIGIT to the beads. To demonstrate that tracer binding was mediated by TIGIT binding, 15 µg of unlabeled anti-TIGIT-DFO was added to the coated beads and incubated for 30-min prior to adding ^89^Zr-αTIGIT. For non-specific tracer binding, there was no antigen added to the beads prior to the addition of the radioconjugate. After one hour, the beads were washed, and the supernatants were collected. Activity of the beads and supernatants were read on a gamma counter.

### Dynamic contrast enhanced (DCE)-MRI

MRIs were performed at 7 Tesla, as previously described^20^, on 6 mice (3 at one week post tumor implant and 3 at two weeks post-implant) to validate tumor presence at one day prior to radiotracer injections. Briefly, under isoflurane anesthesia an anatomical T2-weighted RARE was used to localize the tumors with the following parameters: Repetition time (TR)/Echo time (TE) = 3000/30 ms, FOV of 20 mm, acquisition matrix = 256 × 256, 25 slices with a slice thickness of 0.5 mm, 2 averages, and a RARE factor = 8. For quantitative Dynamic Contrast Enhanced (DCE) MRI, first a T1 map was determined with a variable TR sequence using the following parameters: TR = 400, 842, 1,410, 2,208, 3,554 and 10,000 ms, TE = 7 ms, 9 contiguous 0.5 mm-axial slices centered on the tumor location, RARE factor = 2, NA = 2, 20 × 20 mm FOV and a matrix size of 128 × 128. DCE-MRI was then performed with a series of 100 dynamic RARE images with a temporal resolution of 12.8 s per frame with a TR/TE = 200/5 ms and the same geometry as above. After approximately 128 s (10 baseline images), a bolus of gadobutrol (Gadovist 1.0, Bayer, Wayne, New Jersey, 0.1 mmol/kg, 10 s duration) was manually injected via a tail-vein catheter placed during animal preparation. Pharmacokinetics of the gadobutrol bolus uptake in the tumor was analyzed using the Hoffmann model^20^ using DEC@urLAB^21^.

## Results

**Figure 1:**
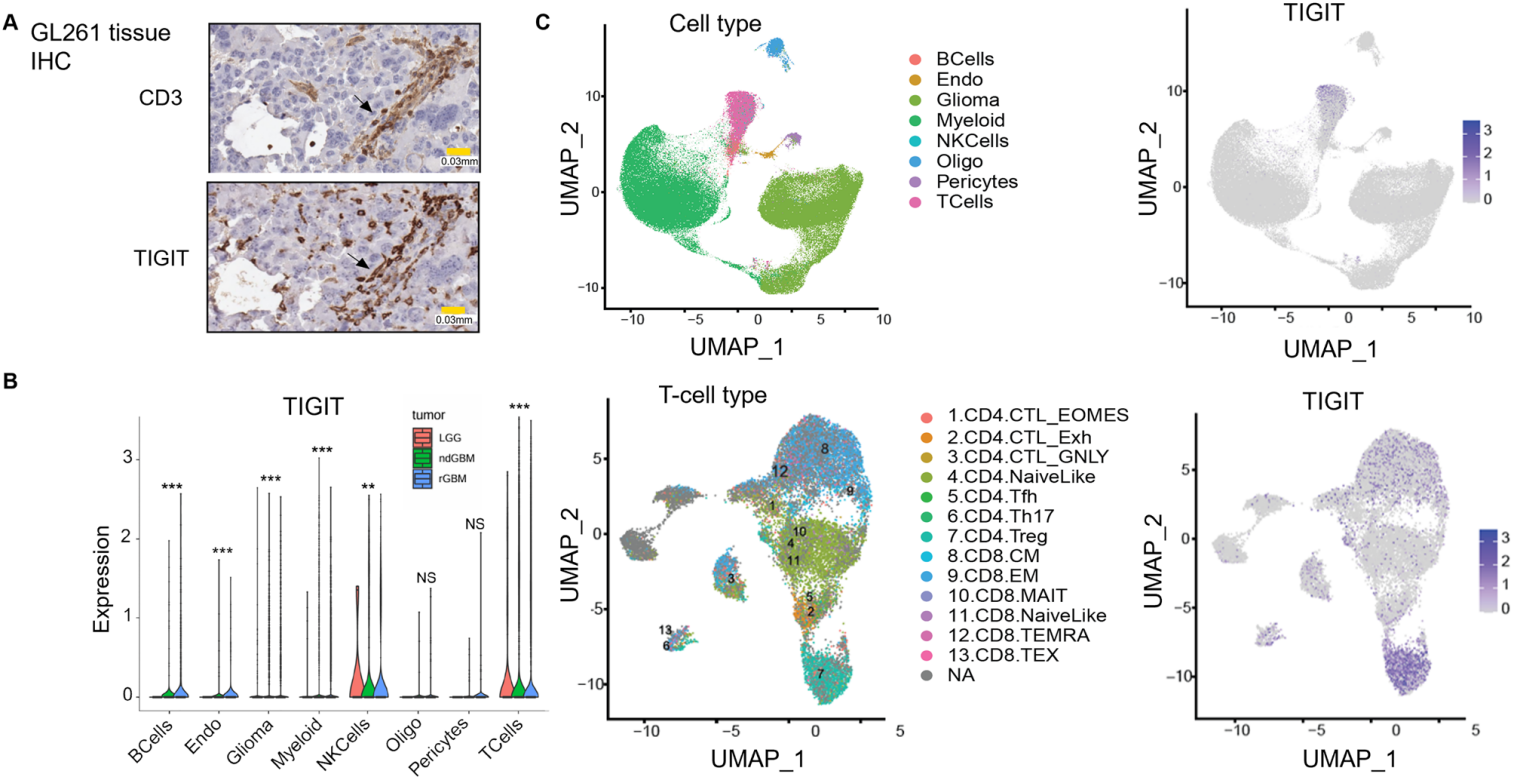
TIGIT is expressed on T-cells and Natural Killer cells in GBM. Histological (**A**) and scRNAseq (**B**,**C**) analyses and assessment of TIGIT expression in murine and patient derived GBM tissues. (**A**) IHC staining of CD3 (left) and TIGIT (right) expression on murine GL261 tissue extracted from 2-week tumor mice. (**B**) Violin plots visualizing TIGIT expression in all glioma cell types among patients with low-grade glioma (LGG), newly diagnosed high-grade glioma (ndGBM), and recurrent high-grade glioma (rGBM); ** denotes p value ≤ 0.01, *** ≤ 0.001    (**C**). Color-coded clustered UMAPs visualizing TIGIT expression in all patient GBM cell types.

To assess the relevance of TIGIT in GBM, TIGIT expression was analyzed by immunohistochemistry (IHC) on murine glioma tissue (GL261 model, Fig. 1A), and by using single-cell (sc)RNAseq data of patient glioma samples (Fig. 1B,C). IHC staining of GL261 tumor tissue revealed areas of CD3 and TIGIT co-localization (Fig. 1A), indicating that TIGIT is expressed among tumor-infiltrating T-cells. TIGIT expression was also observed on CD3^-^ cells. These cells may include NK cells, which are also known to express TIGIT^22–26^. As tumors have been reported to aberrantly express a variety of genes^27^, we evaluated TIGIT expression on GL261 tumor cells by flow cytometry which demonstrated that GL261 tumor cells do not express detectable levels of TIGIT (Supplementary Fig. 1).

We analyzed data from tumor tissue from 18 human gliomas (n = 2 low-grade glioma (LGG), n = 11 newly diagnosed GBM (ndGBM), and n = 5 recurrent GBM (rGBM)) and specifically assessed which cell types express TIGIT^14^. scRNAseq data analysis confirmed almost exclusive TIGIT expression on T- and NK cell populations, and no expression of TIGIT on tumor cells (Fig. 1C). Reclustering of T-cells showed TIGIT expression on multiple T-cell types, including cytotoxic T-cells and T-regulatory cells (Fig. 1C).), TIGIT expression on T- and NK occurred in LGG, newly diagnosed (nd)GBM, and recurrent (r)GBM (Fig. 1B).

**Figure 2:**
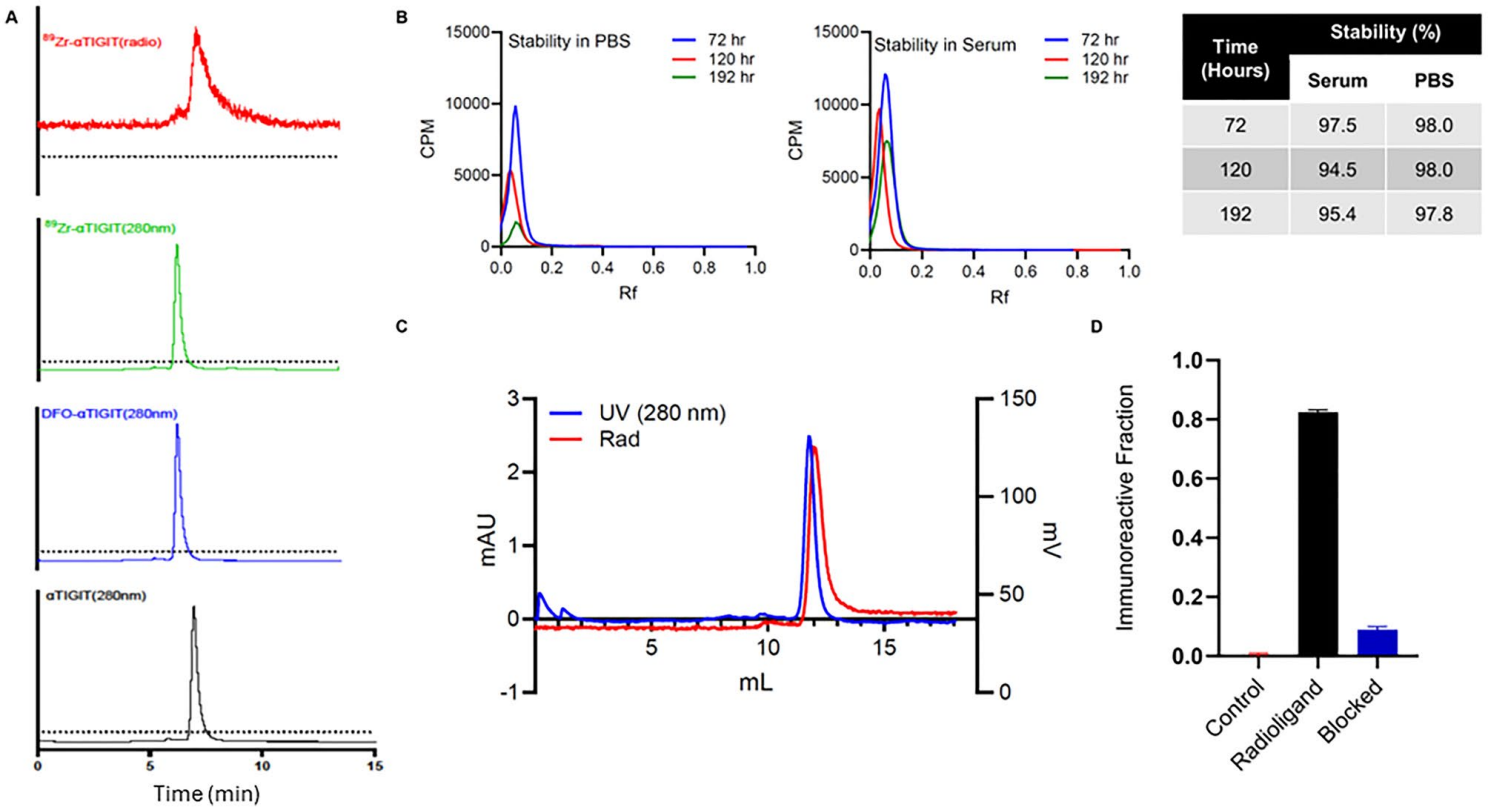
^89^Zr-αTIGIT demonstrates high stability and binding specificity to TIGIT antigen. (**A**) HPLC profiles of generation of ^89^Zr-αTIGIT at room temperature: bioconjugation of TIGIT-DFO followed by ^89^Zr radiolabeling αTIGIT to ^89^Zr-αTIGIT acquired using Agilent Bio SEC-3 column. (**B**) Radio-iTLC stability profiles of ^89^Zr-αTIGIT in PBS (right) and mouse serum(left). (**C**) HPLC profile of ^89^Zr-αTIGIT at 192 h post incubation in PBS acquired using Superdex S200 Increase 10/300 GL SEC column (**D**) Immunoreactivity of 89Zr-αTIGIT, evaluated by a bead-based assay utilizing streptavidin coated magnetic bead. Bead bound activity was counted using Gamma counter to assess the immunoreactivity; Data expressed as ± SD.

We next evaluated whether immunoPET imaging with an anti-TIGIT antibody would be feasible to detect and quantify TIGIT expression in the TME of preclinical GBM. We generated ^89^Zr-αTIGIT using an anti-TIGIT antibody conjugated to p-SCN-bn-DFO, which was subsequently radiolabeled and purified at high radiopurity (> 95%; Fig. 2A). The observed chelator to antibody ratio was 1.5. High stability (> 95%) of ^89^Zr-αTIGIT was observed in both PBS and serum (Fig. 2B,C). To assess the immunoreactive fraction of ^89^Zr-αTIGIT to the TIGIT antigen, a bead-based assay was performed. The unblocked radiotracer bound its target (82.5 ± 0.1% bound) while the antigen-free and antibody-blocked controls exhibited significantly lower tracer bead-bound fractions 0.20 ± 0.1% and 8.90 ± 1.0%, for an immunoreactivity of approximately 73% (Fig. 2D). This data demonstrates that the ^89^Zr-αTIGIT tracer can generate high radiochemical purity and immunoreactivity suitable for molecular imaging of TIGIT.

**Figure 3:**
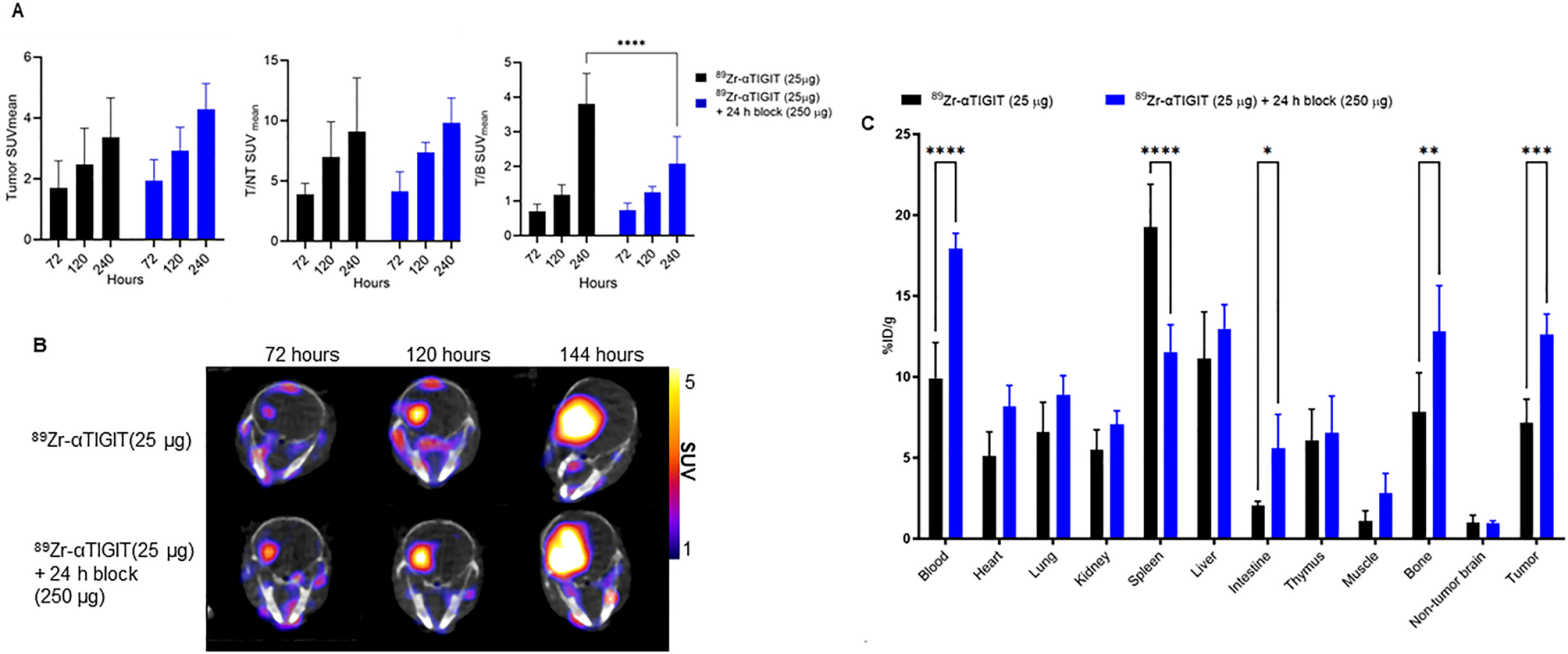
In vivo assessment of ^89^Zr-αTIGIT with and without blocking. Mice bearing GL261 orthotopic tumors (N = 5 per group, two weeks post inoculation) were imaged with PET/CT at 72, 120, and 240-h post-injection of ^89^Zr-αTIGIT (2.3–2.5Mbq/25 µg) alone or ^89^Zr-TIGIT (2.3–2.5 Mb/25 µg) plus unconjugated anti-TIGIT (250 µg) administered 24-h prior to tracer. (**A**) PET image analysis of GL261 tumors' SUVmean and brain tumor to non-tumor ratios of SUVmean (T/NT-SUVmean) and blood (T/B-SUVmean); (**B**) Representative PET/CT images (**C**) Ex vivo biodistribution at ~ 240-h post-injection of tracer. Data analyzed by two-way ANOVA—*p* ≤ *0.05, ≤ **0.01, ≤ ***0.001, and ≤ ****0.0001.

To evaluate ^89^Zr-αTIGIT as a PET tracer, mice bearing GL261 orthotopic tumors were injected intravenously with 25 µg of ^89^Zr-αTIGIT (2.3–2.5 MBq) with or without a blocking dose (24 h-prior with a 10 × unconjugated anti-TIGIT) and PET/CT imaging was performed at multiple time points (72-, 120-, and 240-h) post-tracer injection (Fig. 3B, Supplementary Figure S2). Standardized uptake values (SUV)mean values were assessed along with SUVmean ratios of tumor:normal brain (T/NT) and tumor:blood (T/B). No significant differences were observed between the ^89^Zr-αTIGIT and block group in tumor SUVmean or T/NT-SUVmean ratio at any assessed time point (Fig. 3A). However, a significant difference (p < 0.0001) in T/B-SUVmean was observed at 240-h between ^89^Zr-αTIGIT (3.81 ± 0.87) and the block group (2.08 ± 0.78) (Fig. 3A). Biodistribution analysis at the 240-h timepoint demonstrated no reduction in tumor uptake with the blocking dose, but a significant decrease was observed in the spleen with block (11.63 ± 1.6) as compared to unblock (9.27 ± 2.6) (Fig. 3C). Tumor uptake in the block group (12.6 ± 1.2%ID/g) was significantly greater (*p* = 0.0007) than in the ^89^Zr-αTIGIT group (7.17 ± 1.4%ID/g). Similar results were found in blood with significantly (p < 0.001) more uptake observed in the block group (17.72 ± 1.1%ID/g) compared with the ^89^Zr-αTIGIT group (9.92 ± 2.2%ID/g). The data likely indicates that the spleen is a sink for anti-TIGIT antibody, and blocking of TIGIT antigen in the spleen increases ^89^Zr-αTIGIT in circulation, allowing for the tracer to reach and accumulate within the tumor. This phenomenon has been observed with other immune checkpoint inhibitors, such as anti-PD-L1^28,29^.

**Figure 4:**
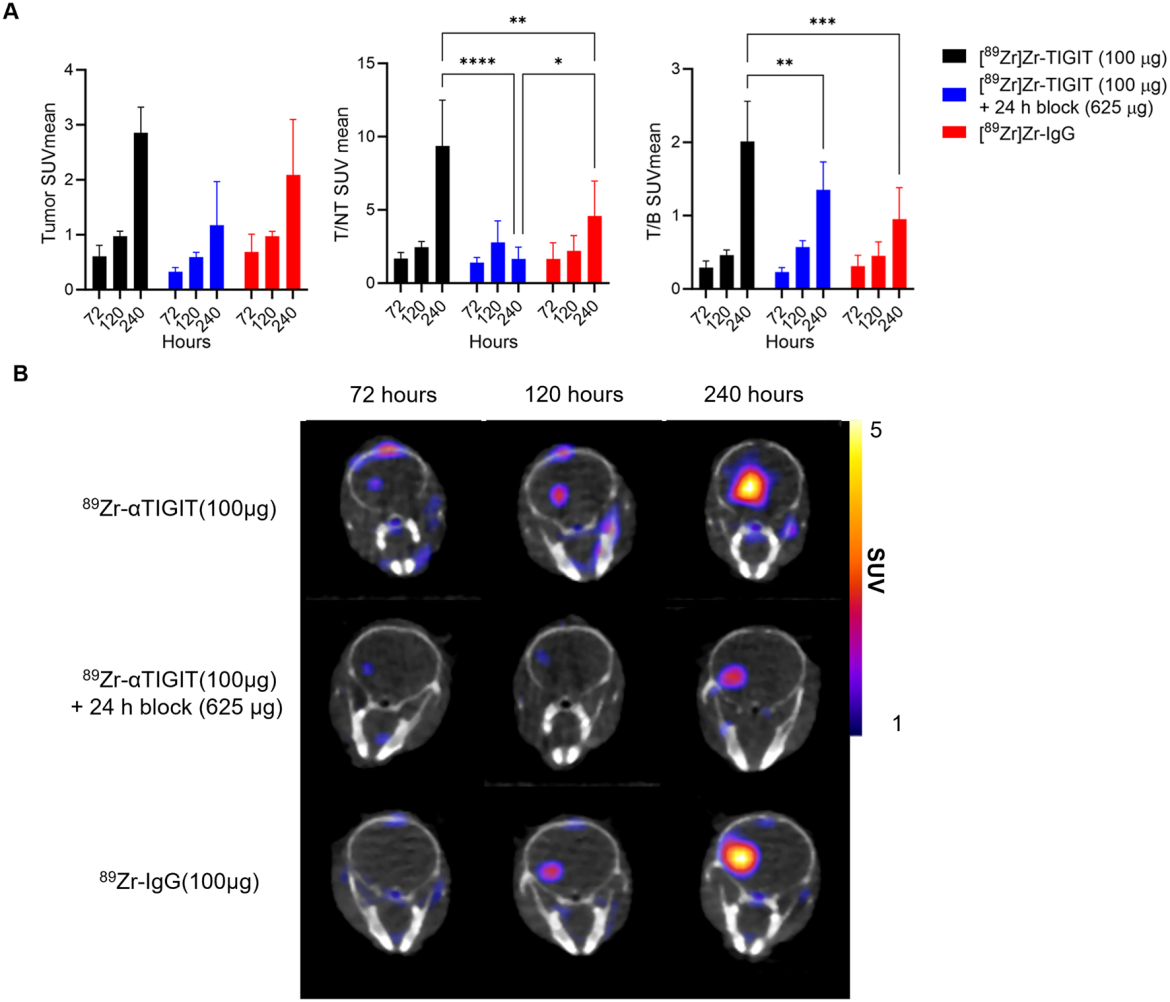
In vivo assessment of ^89^Zr-αTIGIT and ^89^Zr-IgG. Mice bearing GL261 orthotopic tumors (N = 4 per group, one week post inoculation) were imaged by PET/CT at 72, 120, and 240-h post-injection of ^89^Zr-αTIGIT(2.9–3.1Mbq/100 µg) alone, ^89^Zr-αTIGIT (2.9–3.1Mbq/100 µg) plus unconjugated anti-TIGIT (625 µg) administered 24-h prior to tracer, or ^89^Zr-IgG (2.9–3.3 MBq/100 µg). (**A**) PET image analysis of GL261 tumors' SUVmean and brain tumor to non-tumor ratios of SUVmean (T/NT-SUVmean) and blood (T/B-SUVmean) (**B**) Representative PET/CT images. Data analyzed by two-way ANOVA—*p* ≤ *0.05, ≤ **0.01, ≤ ***0.001, and ≤ ****0.0001.

To further evaluate TIGIT-mediated tumor accumulation and overcome peripheral antigen sink, we repeated ^89^Zr-αTIGIT PET studies using a lower molar activity (higher antibody dose; 100 µg) and blocking dose (625 µg unconjugated anti-TIGIT) in mice with 1-week established GL261 tumors (Fig. 4B, Supplementary Figure S3A, S3B). Additionally, a radiolabeled irrelevant (isotype-matched) control antibody was included to assess non-specific uptake and the impact of the enhanced permeability and retention (EPR) effect (Fig. 4B, Supplementary Figure S3C).

PET/CT analysis of SUV_mean_, T/NT-SUV_mean_, and T/B-SUV_mean_ showed significantly higher amounts of ^89^Zr-αTIGIT uptake (2.85 ± 0.46, 9.36 ± 3.34, 2.01 ± 0.55, respectively) compared with mice administered block (2.09 ± 1.01, 1.66 ± 0.79, 1.35 ± 0.38, respectively) or irrelevant antibody control (^89^Zr-IgG1; 1.17 ± 0.89, 4.58 ± 2.39, 0.95 ± 0.43, respectively) at 240-h post-injection, but not at earlier time points (Fig. 4A). The late timepoint allows circulating ^89^Zr-αTIGIT to clear as suggested by the decreasing SUVmean in the blood and heart (Supplementary Figure S4). Furthermore, mice in the block group demonstrated significantly lower blood (vena cava and heart) uptake (SUV_mean_) compared with mice from the ^89^Zr-αTIGIT alone group at the 72 and 120-h timepoints (Supplementary Figure S4), suggesting that selective binding to circulating immune cells may be occurring. This may also explain the higher tracer SUVmean over time as these circulating immune cells may accumulate within the tumor over time. ^89^Zr-αTIGIT showed a bone marrow SUVmean of 0.43 ± 0.25 with blocking and 0.78 ± 0.16 without blocking at 240 h with *p* ≤ 0.02. No significant increase or decrease in uptake was observed at 72 and 120 h with blocking. No significant differences were observed between ^89^Zr-αTIGIT and ^89^Zr-IgG.

**Figure 5:**
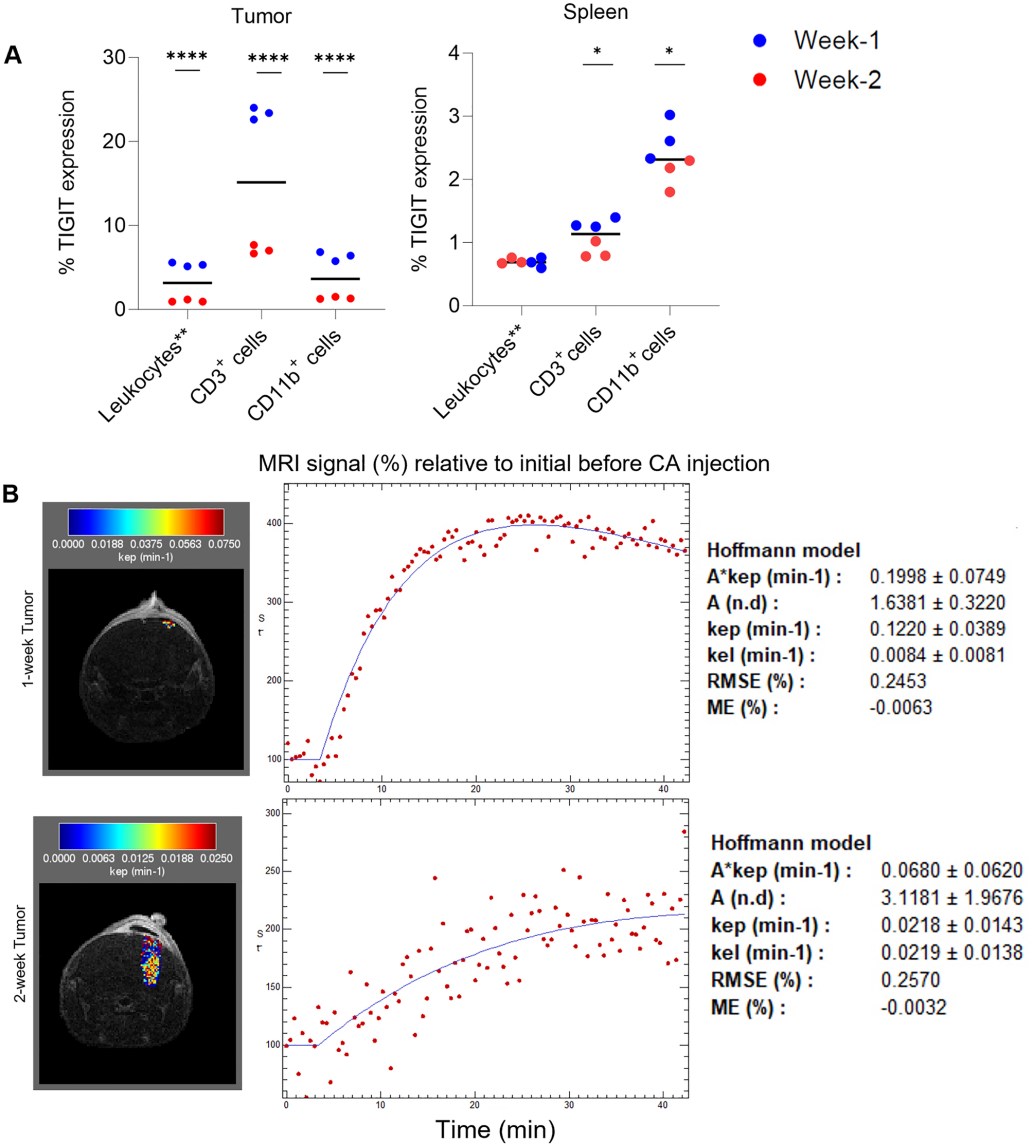
TIGIT PET signal is positively correlated with tumor progression. (**A**) Flow cytometry results illustrating TIGIT expression among total FSC/SSC-gated leukocytes (leukocytes**), CD3, and CD11b cell subsets in tumor (left) and spleen (right) tissue. (**B**) kep color maps for the tumor ROIs were overlaid onto greyscale anatomical MRI images from representative animals (Left Panels). Representative MR signal time course with the fitted Hoffman model for tumors at one week and two weeks post implantation (Right Panels).

Flow cytometry and dynamic contrast enhanced (DCE) MRIs were performed to evaluate TIGIT expression and precisely measure differences in tumor size and levels of washout between mice (Fig. 5A,B). By flow cytometry analysis TIGIT expression was found to be significantly higher in CD3^+^ and CD11b^+^ tumor-infiltrating lymphocytes (TILs) from two-week tumors versus those with one-week tumors. (23.3% vs. 7.1% CD3^+^ *p* < 0.0001 and 7.3% vs 1.4% CD11B^+^ *p* < 0.0001), (Fig. 5A and Supplementary Figures S5, S6). CD3^+^ and CD11B^+^ splenocytes showed a similar trend of TIGIT expression, although less pronounced compared with the tumor (1.3% vs 0.86% CD3^+^ *p* = 0.0456 and 2.7% vs 2.1% CD11B^+^ *p* = 0.0117), (Fig. 5A and Supplementary Figures S5, S6). DCE MRI analysis revealed a reduced level of washout in mice bearing larger two-week tumors compared to mice bearing one-week tumors, with the mean 2-week tumor k_ep_ = 0.028 ± 0.008 vs. 1-week tumor k_ep_ = 0.104 ± 0.015, (Fig. 5B).

## Discussion

Immunotherapy has seen great success in the treatment of some malignancies residing outside the CNS; however, in GBM, immunotherapy clinical trials have shown limited efficacy. It is believed that the failure of immunotherapy trials in gliomas is due, in part, to the highly immunosuppressive nature of the TME^30^. To improve immunotherapy outcomes for patients with glioma a more thorough understanding of immune checkpoints in the TME is essential.

TIGIT, an immune checkpoint receptor, serves as an inhibitor of anti-tumor immune responses^31^. Quantification of TIGIT within the glioma TME has the potential to assess an aspect of immunosuppression to select glioma patients for anti-TIGIT immunotherapy. Clinical trials are emerging that evaluate the efficacy of multiple immune checkpoint inhibitors for glioma, including blocking of TIGIT. We focused on the development of a TIGIT-targeted immunoPET agent to allow quantification of TIGIT expression in gliomas.

We previously examined TIGIT expression in 28 primary human gliomas by scRNAseq and found that CD11b^+^ TAMs, including myeloid-derived suppressor cells, expressed genes for PD1, PD-L1, and TIGIT ligands^9^. The overall findings in that study supported the idea that TIGIT and PD-1 pathways contribute to immunosuppression and tumor aggression in glioma^13^. Here we analyzed a separate scRNAseq data set including LGG, ndGBM and rGBM to extend these findings. We show that TIGIT expression is detectable on NK cells and T-cells in LGG (as well as GBM) by scRNAseq. Further reclustering of T-cells identified that TIGIT is predominantly expressed on CD4^+^ Tregs and specific CD8 T-cells including those with an effector memory and central memory phenotypes. However, TIGIT expression was also identified at low frequencies within other T-cell populations. These data provide further support for the role of TIGIT in gliomas and as an imaging target.

To develop a molecular imaging agent for TIGIT, we utilized an anti-TIGIT antibody conjugated with DFO. The anti-TIGIT antibody used in this study (clone 1G9) is the same as we previously used to show therapeutic benefits with multiple treatments and a higher dose^9^. We evaluated ^89^Zr-αTIGIT in a murine model of GBM. By comparison to blocking and irrelevant antibody controls, the in vivo immunoPET imaging (Fig. 4) indicated that ^89^Zr-αTIGIT can specifically detect TIGIT-expressing cells in a murine model of GBM, at late timepoints. However, while significant, the magnitude of difference in SUVmean between ^89^Zr-αTIGIT and the ^89^Zr-IgG groups was minimal, and the uptake of ^89^Zr-IgG was high. While this nonspecific uptake was high, it should be noted that the ^89^Zr-IgG is not expected to experience antigen sink, which may result in higher tumor uptake relative to the targeted tracer, which would experience antigen sink. Additionally, we performed DCE-MRI to assess washout by Kep, which is the washout of contrast from the tumor compartment into plasma. 1-week established tumors demonstrated high washout compared with 2-week established tumors, suggesting at this time point lower non-specific retention. At the early timepoints (72- and 120-h) TIGIT-specific uptake was not significantly different compared with uptake in mice receiving a blocking dose, highlighting another confounding factor in using ^89^Zr-αTIGIT. The challenges observed with ^89^Zr-αTIGIT in our model may be, in part, attributed to suboptimal dosing. Future experiments focused on optimizing dose, including the use of an irrelevant tracer to optimize signal to noise, and a blocking experiment with high dose of unconjugated Ab, may improve the utility of ^89^Zr-αTIGIT. Additionally, future studies may evaluate the use of targeting molecules with shorter half-lives than IgG, such as antibody fragments and peptides, which may allow imaging at early time-points. Additionally, therapeutic interventions, such as those that increase TIGIT-expressing T-cell numbers, which may enhance total TIGIT levels within GBM, may be more suitable for detection of ^89^Zr-αTIGIT via immunoPET. Overall, ^89^Zr-αTIGIT immunoPET is feasible and demonstrates specific binding in a murine model of GBM, however, the utility is greatly limited by multiple factors.

### Supplementary Information

Supplementary Figures.

## Data Availability

The raw single cell sequencing data analyzed in this study is publicly available with no restrictions through the GEO series, GSE182109, GEO Accession viewer (nih.gov).

## Abbreviations

GBM: Glioblastoma
TIGIT: T-cell immunoreceptor with Ig and ITIM domain
NK cell: Natural killer cell
Treg: Regulatory T-cell
APC: Antigen presenting cell
PET: Positron emission tomography
DMEM: Dulbecco’s modified eagle medium
scRNAseq: Single-cell RNA sequencing
LGG: Low-grade glioma
ndGBM: Newly-diagnosed glioblastoma
rGBM: Recurrent glioblastoma
UMAP: Uniform manifold approximation and projection
TME: Tumor microenvironment
SUVmean: Mean standard uptake value
SUVmax: Max standard uptake value
