# Supplementary material for: ImmunoPET imaging of TIGIT in the glioma microenvironment
Source: Sci Rep. 2024 Mar 4;14:5305. doi: 10.1038/s41598-024-55296-y (PMC10912309; doi:10.1038/s41598-024-55296-y)
Supplement: Supplementary file 1 — Supplementary Figures. [file 41598_2024_55296_MOESM1_ESM.pdf]

Title: ImmunoPET imaging of TIGIT in the glioma microenvironment

Sarah R. Vincze[1#], Ambika P. Jaswal[1#], Stephen C. Frederico[1#], Michal Nisnboym[1,2], Bo Li[1], Zujian Xiong[1], ReidAnn E. Sever[1], Chaim T. Sneiderman[1], Mikayla Rodgers[5], Kathryn E. Day[3], Joseph D. LaToche[3], Lesley M Foley[3], T. Kevin Hitchens[3,7], Robin Frederick[3], Ravi B. Patel[4], Costas G. Hadjipanayis[1], Itay Raphael[1], Jessie R. Nedrow[3,4*], W. Barry Edwards[5*], and Gary Kohanbash[1,6*]

[1]Department of Neurological Surgery, University of Pittsburgh School of Medicine, Pittsburgh, PA, USA
[2]Department of Neurology, Tel-Aviv Sourasky Medical Center, Tel-Aviv University, Israel
[3]In Vivo Imaging Facility, University of Pittsburgh Medical Center, UPMC Hillman Cancer Center, Pittsburgh, PA, USA
[4]Department of Radiology, University of Pittsburgh Medical Center, Pittsburgh, PA, USA
[5]Department of Biochemistry, University of Missouri, Columbia, MO, USA
[6]Department of Immunology, University of Pittsburgh, Pittsburgh, PA, USA
[7]Department of Neurobiology, University of Pittsburgh, Pittsburgh, PA, USA

# Co-first authors

***Corresponding author:**

Jessie R. Nedrow, Department of Radiology, University of Pittsburgh.
5117 Centre Avenue, Suite G. 17b, Pittsburgh, PA 15213, USA
Phone Office: 412.623.7239; Email: nedrowj@upmc.edu.

Wilson B. Edwards. Department of Biochemistry. University of Missouri.
222 Schweitzer Hall, Columbia, MO 65211.
Phone: 573.882.4845; E-mail: wbe59z@missouri.edu.

Gary Kohanbash, Department of Neurological Surgery, University of Pittsburgh, Children's Hospital of Pittsburgh of UPMC, 7128 Rangos Research Building, 530 45th Street, Pittsburgh, Pennsylvania 15201, USA. Phone: 412.692.9456; Email: gary.kohanbash2@chp.edu

Supplementary Figure S1

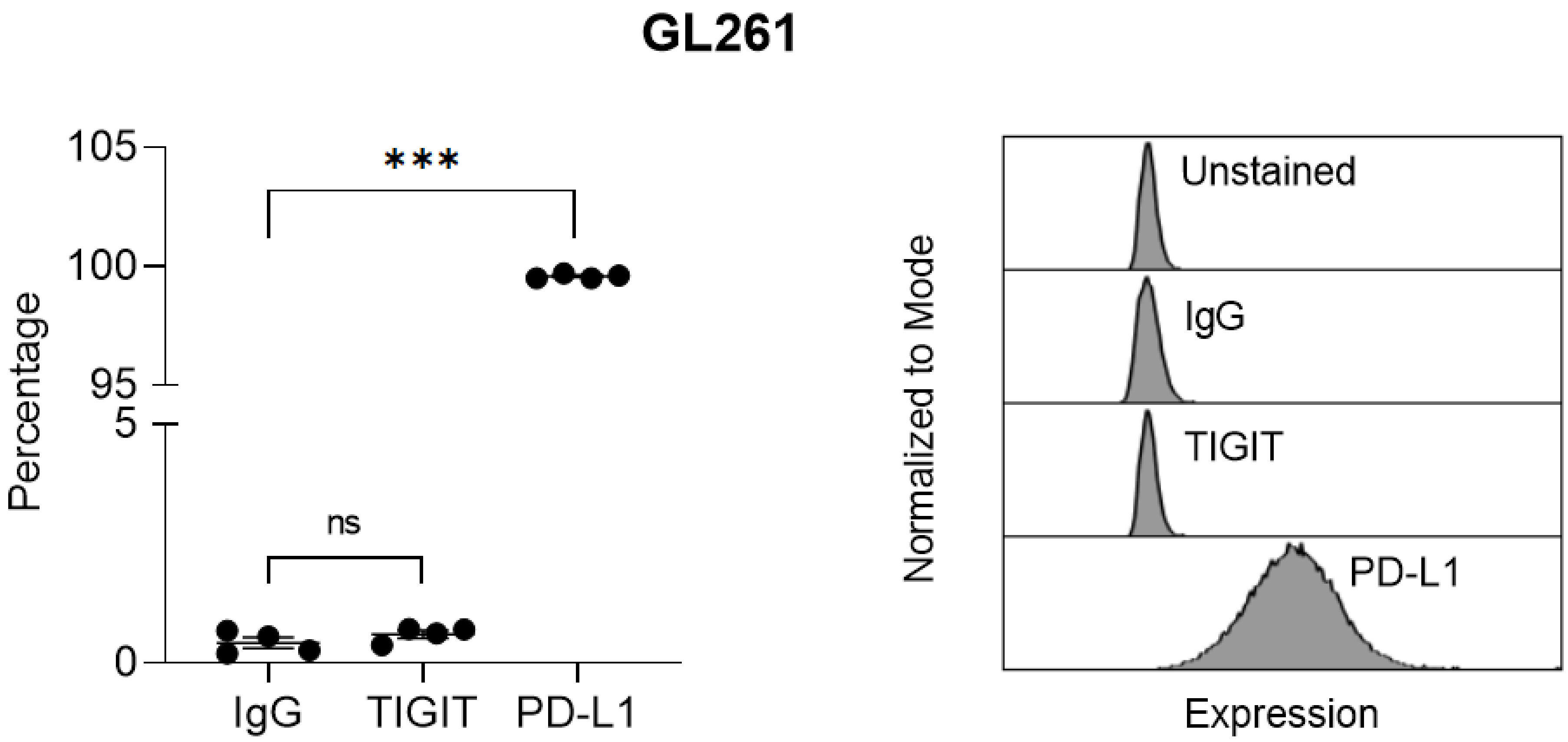

Supplementary Figure S1. Mean expression percentages of TIGIT and the IgG isotype control were comparable to the unstained control and in sharp contrast to the positive control, PD-L1, (IgG=0.42%, TIGIT=0.6%, PD-L1=99.6%, indicating that TIGIT is not expressed on GL261 cells.

Supplementary Figure S2

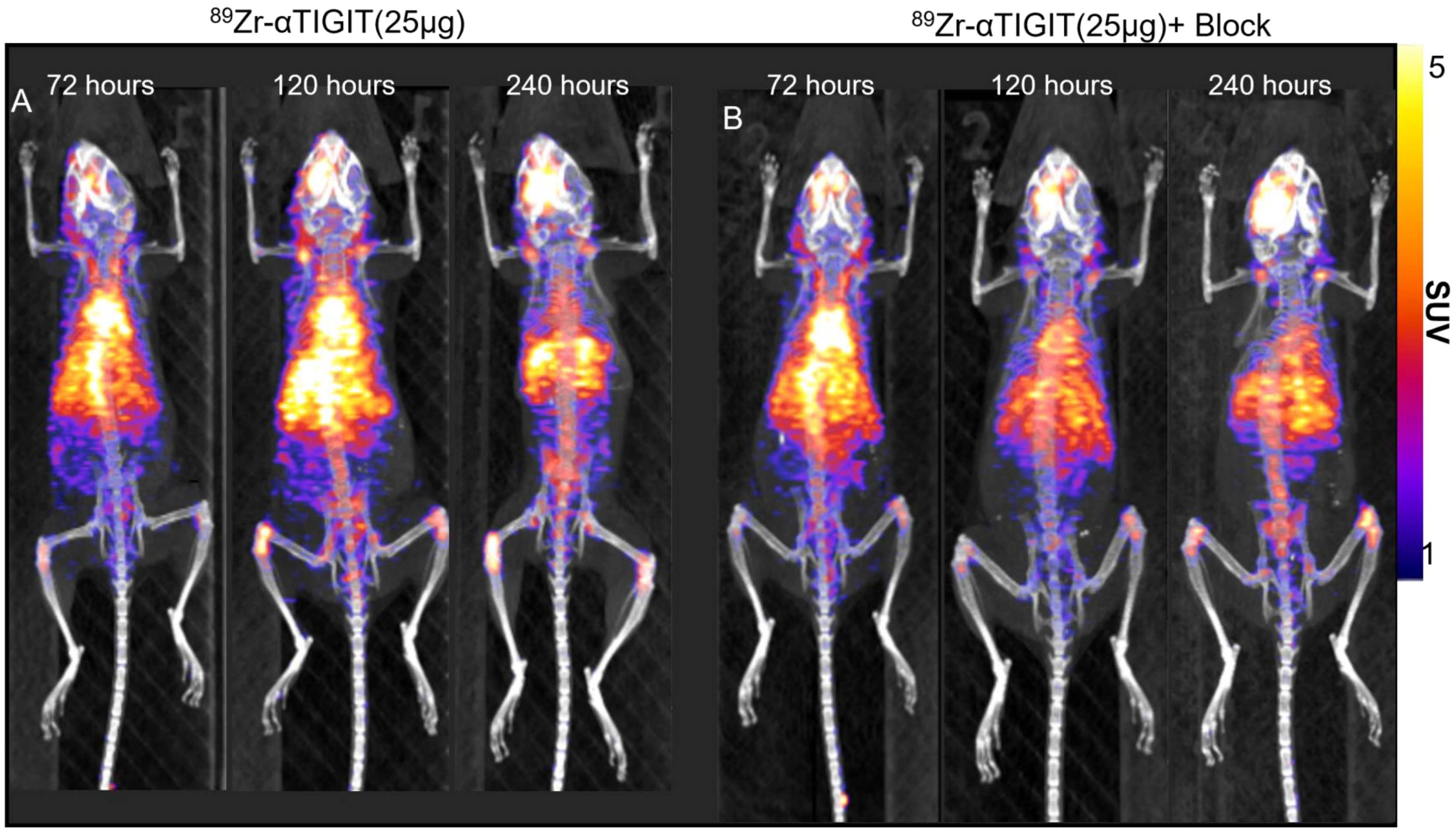

Supplementary Figure S2. Whole body PET/CT images at 72,120 and 240-hours post-injection of ^{89}Zr-αTIGIT(2.5-2.7MBq/25µg) alone and ^{89}Zr-αTIGIT(2.5-2.7MBq/25µg) plus unconjugated anti-TIGIT antibody(250µg) administered 24-hours prior to tracer.

Supplementary Figure S3

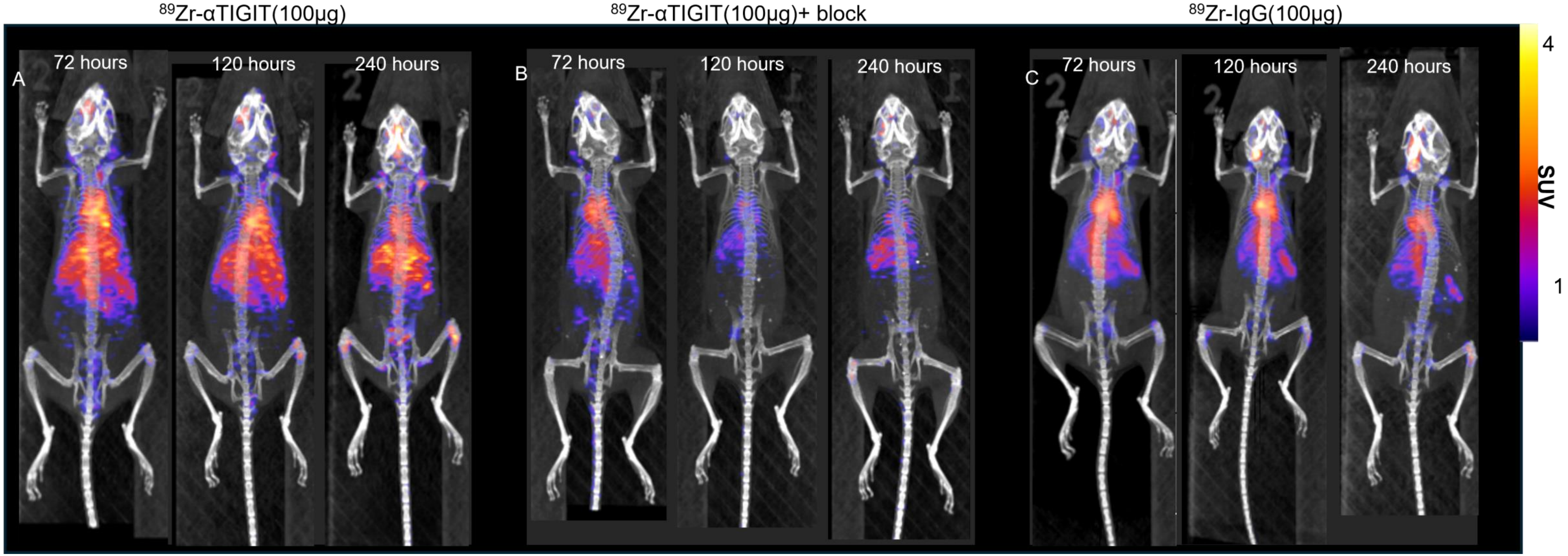

Supplementary FigureS3: Whole body PET/CT images of ^{89}Zr-TIGIT at 72,120 and 240-hours post injection of (A) 2.9-3.3MBq ^{89}Zr-αTIGIT with 100µg (B)2.9-3.3MBq ^{89}Zr-αTIGIT with 625µg unlabeled anti-TIGIT antibody injected 24 hours prior radiotracer injection (C) 3.1-3.5MBq ^{89}Zr-IgG.

Supplementary Figure S4

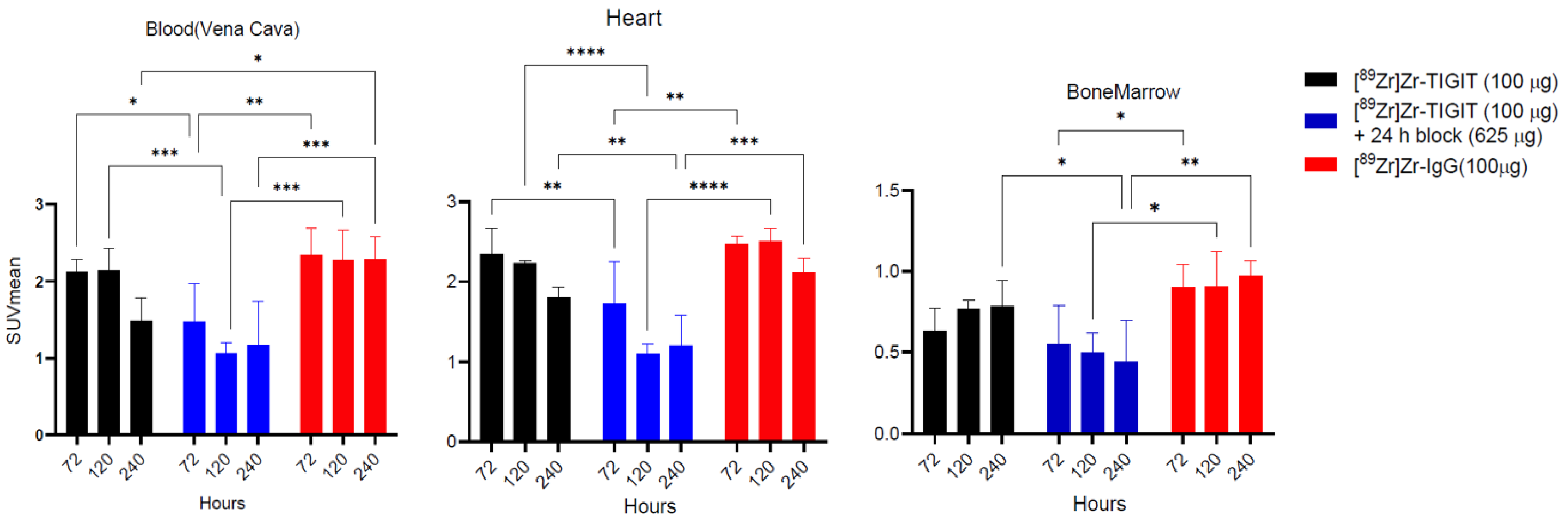

Supplementary Figure S4. Bar graph demonstrating variation of SUV mean for Blood, heart, Bone M arrow for ^{89}Zr-αTIGIT(100µg, black), ^{89}Zr-αTIGIT(100µg with 625µg block, blue) and ^{89}Zr-αTIGIT (100µg) 72, 120, 240 hours post radiotracer injection in GL261 glioma models one week post tumor inoculation. Data represented as ±SD

Supplementary Figure S5

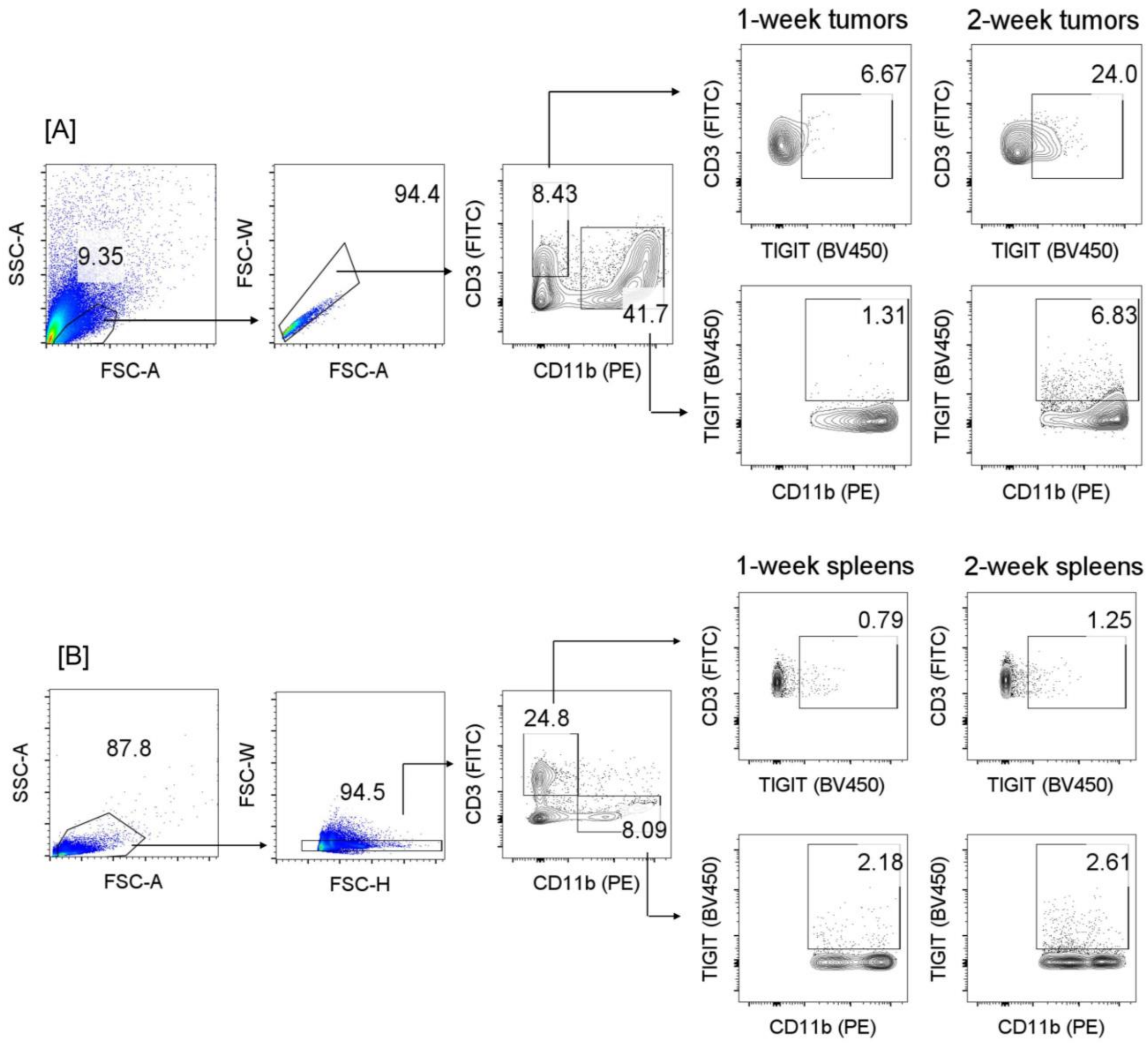

Supplementary Figure S5. Gating strategies used for flow cytometry data analysis of tumor (A) and spleen (B) samples.

Supplementary Figure S6

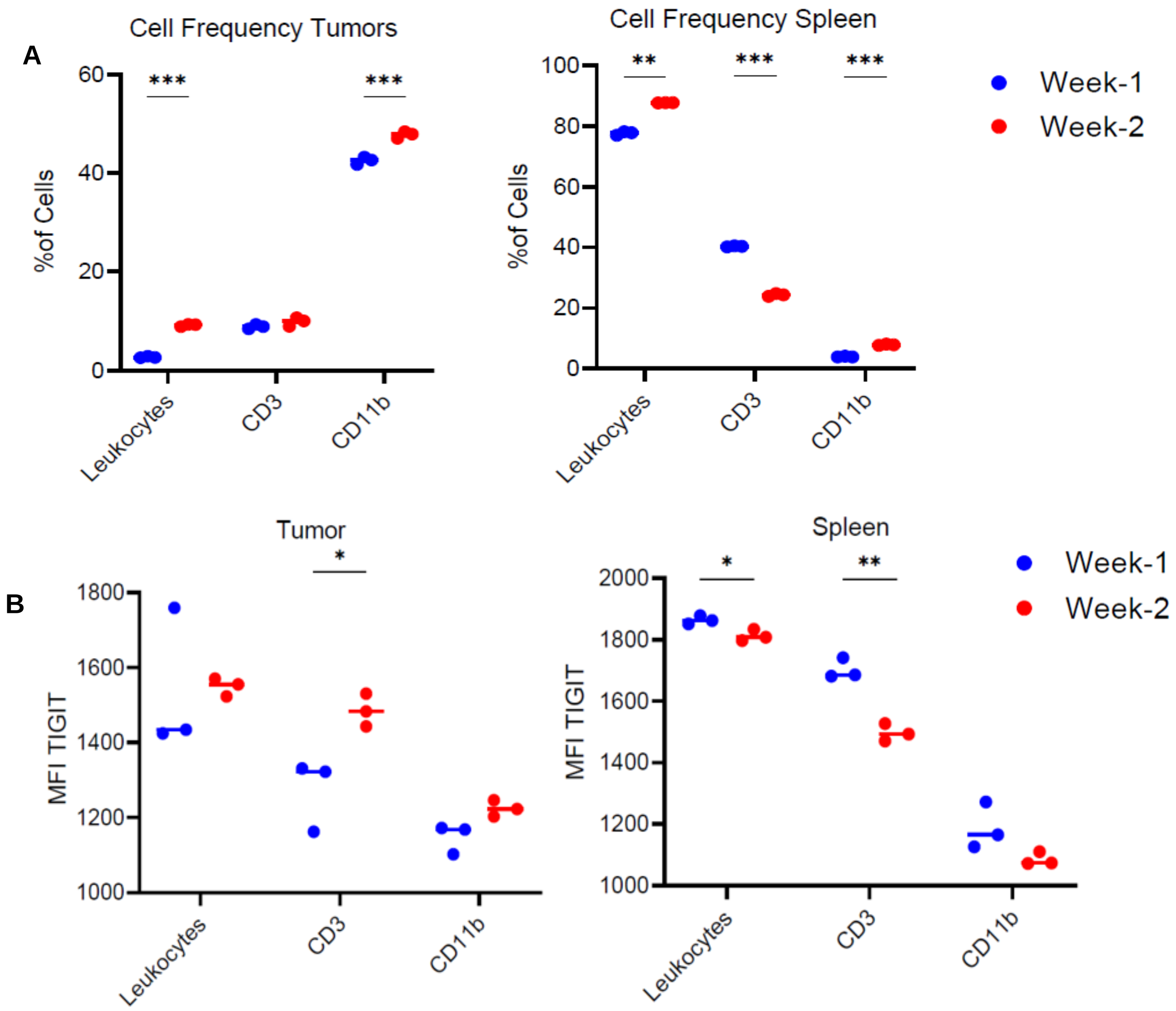

Supplementary Figure S6. MFI and % values for TIGIT in GL261 gliomas. ((A) Percentage of total Leukocytes, CD3 and CD11b positive cells in week-1 and week-2 tumor and spleen samples by Flow cytometry and (B) Flow cytometry analysis of MFI in week-1 and week-2 tumor and spleen samples
